# Hybrid Albumin-Decorated Lipid-Nanocarrier-Mediated Delivery of Polyphenol-Rich *Sambucus nigra* L. in a Potential Multiple Antitumoural Therapy

**DOI:** 10.3390/ijms252011206

**Published:** 2024-10-18

**Authors:** Robert Tincu, Mirela Mihaila, Marinela Bostan, Daniela Istrati, Nicoleta Badea, Ioana Lacatusu

**Affiliations:** 1 Faculty of Chemical Engineering and Bioengineering, National University of Science and Technology Politehnica Bucharest, Polizu No 1, 011061 Bucharest, Romania; robert.tincu@ccocdn.ro (R.T.); daniela.istrati@upb.ro (D.I.); nicoleta.badea@upb.ro (N.B.); 2 “C. D. Nenitzescu” Institute of Organic and Supramolecular Chemistry of the Romanian Academy, 202B Splaiul Independentei, 060023 Bucharest, Romania; 3 Stefan S. Nicolau Institute of Virology, Mihai Bravu Street No 285, 030304 Bucharest, Romania; marinela.bostan@virology.ro; 4 Faculty of Pharmacy, Titu Maiorescu University, Bd. Gh. Sincai No. 16, 040314 Bucharest, Romania; 5 Department of Immunology, Victor Babes National Institute of Pathology, 99-101 Splaiul Independetei, 050096 Bucharest, Romania

**Keywords:** *Sambucus -nigra*, protein–lipid nanocarriers, antioxidant effect, phytochemical entrapment, apoptosis process, cell cycle arrest

## Abstract

The current research attempted to address the suitability of bioactive *Sambucus nigra* extract entrapped in albumin-decorated nanostructured lipid carriers (NLCs) as a promising “adjuvant” in improving tumour penetration for multiple antitumour therapy. The new hybrid albumin-decorated NLCs were characterised based on, e.g., the particle size, zeta electrokinetic potential, *SambucusN* entrapment efficiency, and fluorescence spectroscopy and tested for different formulation parameters. The antioxidant activity of NLC-*SambucusN* was significantly enhanced by a bovine serum albumin (BSA) polymer coating. According to the real-time cell analysis (RTCA) results, NLC-I–*SambucusN*–BSA behaved similarly to the chemotherapeutic drug, cisplatin, with cell viability for LoVo tumour cells of 21.81 ± 1.18%. The new albumin–NLC–*SambucusN* arrested cancer cells in G1 and G2 cycles and intensified the apoptosis process in both early and late phases. An advanced induction, over 50% apoptosis in LoVo colon cells, was registered for 50 μg/mL of NLC-II-*SambucusN*-BSA, a fourfold increase compared to that of untreated cells. RTCA and flow cytometry results showed that concentrations of the hybrid NLC–*SambucusN* up to 50 μg/mL do not affect the proliferation of normal HUVEC cells. This approach provides insightful information regarding the involvement of phytochemicals in future therapeutic strategies. Albumin-decorated NLCs can be considered a noteworthy strategy to be connected to antitumour therapeutic protocols.

## 1. Introduction

Bovine serum albumin (BSA) is a biopolymer extensively applied to fabricate a large variety of nanocarriers due to its excellent biosafety and biocompatibility, making it a non-immunogenic and stable plasma–protein [1]. Of the currently used proteins for decorating nanocarriers, albumin stands out due to its notable benefits, e.g., the ability to solubilise various drugs, its excellent aqueous solubility, and stability at pH 4–9 [2]. Owing to the surface location of charged groups, which are rich in negative and positive charges, albumin is a versatile protein used to develop innovative drug-delivery systems for cancer therapeutics [3]. As an endogenous protein, albumin endows nanoparticles with prolonged circulation in the blood, while the hepatobiliary system can efficiently remove albumin-based nanoparticles [4]. Furthermore, albumin is one of the efficient biomimetic molecules with a cancer-targeting ability [5] because it can bind the gp60 receptor and SPARC protein, which are overexpressed in several cancer cells [6]. Because tumour cells predominantly internalise albumin as nutrition through a pathway mediated by the glycoprotein gp60 (albondin), albumin can activate the corresponding receptor in the tumour vasculature [6]. Albumin enters via gp60-mediated endocytosis, followed by a combination with SPARC. SPARC is a glycoprotein from the extracellular matrix that enhances the accumulation of albumin inside the tumour by providing mediated transport to the subendothelial space [7]. Thus, albumin is an ideal protein that can serve as an excellent ligand endow nanocarriers with unique properties; the drug release from albumin nanocarriers can be easily regulated because cancer cells efficiently metabolise albumin.

The nanoparticles (NPs) used as carriers for cancer therapeutics may be of several types, including protein-based NPs (e.g., albumin NPs) [2], metallic NPs, polymer-based NPs (e.g., polycaprolactone NPs, poly lactide–co–glycolide NPs, chitosan NPs, etc.) [8], lipid-based NPs (e.g., liposomes, solid lipid nanoparticles—SLNs, and nanostructured lipid carriers—NLCs) [9], etc. For instance, cholesterol-based lipid NPs decorated with albumin were synthesised for the delivery of quinacrine to enhance dose-dependent behaviour against A-549 lung cancer cells through the inhibition of the Nrf2 signal pathway and arrest of the cell cycle mechanism [10]. Albumin-conjugated doxorubicin nanocomposites were studied to overcome the multidrug resistance of breast cancer cells [11]. Combined therapy using neratinib and silibinin administered via albumin-based nanocarriers was also explored to target breast cancer [12]. Other amphotericin–BSA-loaded nanoemulsions and microemulsions for oral delivery were recently reported by Marcelino et al. [13]. Among all these nanocarriers, albumin-based lipid nanoparticles have gained much more attention in cancer therapy because they combine the biocompatibility and mucoadhesivity of lipids and endogen proteins while avoiding the immunogenicity and biological instability exhibited by most organic nanoparticles [14]. In order to reach systemic circulation after oral administration, the drugs have to overcome many barriers, including enzymatic, sulfhydryl (the thiol/disulphide exchange reaction in the gastrointestinal tract), mucus, and epithelial barriers [15]. Lipid nanoparticles, such as SLNs/solid lipid nanoparticles and NLCs/nanostructured lipid carriers, are robust nanocarriers for therapeutic actives that can be surface nanoengineered with endogen protein to facilitate prolonged systemic circulation; for instance, the formation of an albumin crown around NLCs influences the fate and performance of lipid nanoparticles in biological fluids. Most nanomedicines in clinical cancer trials are liposomal formulations, but there has been a noteworthy increase in the number of other lipid nanoparticles used. Although albumin-based SLNs and NLCs are able to show a lot of promising advantages and benefits, there are still only a few studies addressing albumin in relation to its associations with SLNs and NLCs [16,17]. In this study, bovine serum albumin-decorated nanostructured lipid carriers were constructed for the delivery of polyphenol-rich *Sambucus nigra* extract to offer a potential dual therapy against breast, colon, and ovarian cancer cells. The presence of abundant functional groups, like carboxyl and amino groups, in the structure of the albumin offers encouraging possibilities for the surface modification of conventional NLCs. Besides these, BSA provides high-affinity hydrophobic binding sites, which can be used to bind ligands via covalent or non-covalent bonds [1], e.g., electrostatic adsorption or surface-coating techniques may be used for the non-covalent attachment of ligands [4]. The surface modification of NLCs with albumin polymers is necessary to alter the NLCs’ surface properties, to modify the pharmacokinetic behaviour, to improve the stability, to prolong the circulation half-life, and to enhance the targeting potential of the delivery system [18]. There is a great expansion of targeting strategies in the field of phytopharmaceuticals for the delivery of herbal bioactives and extracts [19]. This emerging plant revolution has been directed towards the development of NLCs that are capable of co-entrapping poorly soluble herbal bioactives and plant extracts to improve their bioavailabilities and therapeutic efficacies [20]. The application of pharmaceutical phytonanotechnology for plant extracts is gaining tremendous growth and interest among scientists [21]. Our research group applied the NLC approach to exploit the therapeutic potential of reliable and effective bioactive plant extracts [22,23,24].

*Sambucus nigra* extract (*SambucusN*) is a rich source of bioactive compounds, mainly polyphenols and anthocyanins, which primarily occur as glycosides and acyl glycosides in elderberry. The polyphenols (phenolic acids and flavonoids) in *SambucusN* are chlorogenic acid, neochlorogenic acid, gallic acid, caffeic acid, vanillic acid, coumaric acid, quercetin, isoquercetin (quercetin-3-glucoside), rutin (quercetin-3-rutinoside), syringetin, kaempferol-3-rutinoside, kaempferol-3-glucoside (astragaline), etc. [25,26]. Besides these bioactives, *Sambucus nigra* contains anthocyanins, especially cyanidin-3-glucoside and cyanydin-3-sambubioside, and small amounts of procyanidins with a low condensation degree, i.e., epicatechin and catechin [25]. Thanks to these bioactives, *SambucusN* is known as a traditional remedy for various kinds of ailments and diseases, its medicinal potential being present in several studies, i.e., antiviral activity [27], antibacterial effect [25], diabetes and metabolic dysfunctions [28], antidepressant potential [29], and antioxidant and antitumour activity [30]. The main bioactivity of *SambucusN* is linked to its ability to scavenge free radicals and prevent lipid peroxidation, which significantly affects its health-promoting properties. It contributes to mitigating injuries caused by oxidative damage that has been linked to several diseases, including cancer. The research results emphasised the valuable cytotoxic and antitumour properties of *SambucusN* extract and their main bioactive, i.e., quercetin, kaempferol, rutin [31,32], chlorogenic and p-coumaric acids [33], and anthocyanins [34], as well as tannins against bladder [35], breast [36], pancreatic [37], colon [38], and ovarian [39] cancer cells. The antitumour effects exerted by bioactives from *SambucusN* extract derive mainly from inducing apoptosis dependent on the regulation of radical oxygen species levels [31] by modulating Akt and NF-κB pathways [40], inhibiting the proliferation and migration of these cells via interplay between flavonoid and miRNA in the regulation of apoptosis [39], and increasing the expression of beta-galactosidase [41] and invasion of cancer cells by targeting the p53 protein [42]. Recently, studies found that anthocyanins from *SambucusN,* i.e., cyanidin 3-*O*-β-D-glucoside, target the tumour lipid membrane [43]. Such interactions of anthocyanins–lipid membranes result in structural changes to the lipid membranes, affecting the resultant biological functions, which then trigger and mediate the bioactivities of anthocyanins [44]. In addition to the cytotoxic effect on cancer cells, according to a study by Banach et al., *SambucusN* extract may enhance the immune response, supporting the body’s response to cancer [34]. All these reported results confirm the chemopreventive and therapeutic effect of *SambucusN* extract and can support an alternative oncological therapy with limited side effects. Unfortunately, *SambucusN*, like most bioactive phytocompounds, has a poor bioavailability that hinders the effective manifestation of its therapeutic potential. The encapsulation approach for *SambucusN* can represent a solution, but we found limited results that address the challenge underlying the development of effective nanodelivery systems for this bioactive extract. Mendes et al. proposed nanophytosome formulations for anthocyanins-enriched extract/*Sambucus nigra* in order to modulate mitochondrial dysfunctions in degenerative brain pathologies like Parkinson’s and Alzheimer’s diseases [45].

Considering the world’s growing interest in natural health-promoting actives, the current study focused on investigating the suitability of bioactive *Sambucus nigra* extract entrapped in hybrid albumin-decorated–nanostructured lipid carriers, designed to improve tumour penetration and targeting for a multiple-antitumour therapy. The lipid nanocarriers and hybrid protein–lipid nanocarriers (BSA-decorated NLC) host two categories of lipophilic core, one consisting of the natural lipids MSG, coconut butter, and sage oil and another consisting of synthetic oil (menthyl laurate) associated with coconut butter and glycerol monostearate. To support our hypothesis, conventional NLC and new polymer-decorated NLC-*SambucusN* (NLC loaded with *Sambucus nigra* extract) were characterised and tested for different formulation parameters, stability, and in vitro antioxidant and antitumour potential. The viability, levels of cell-cycle arrest, and apoptosis were determined on different tumour cell lines, i.e., MCF-7 breast, LoVo colon, and SKOV-3 ovarian adenocarcinoma cells, and their performance was compared to conventional chemotherapy drugs, doxorubicin and cisplatin. To our knowledge, this study is the first to explore the combinatorial potential of *Sambucus nigra* and albumin-decorated–nanostructured lipid carriers as new sustainable nanocarriers to support antitumour targeted therapy, particularly for the synergistic therapy of colon, ovarian, and/or breast cancer.

## 2. Results and Discussion

### 2.1. Particle Size, Surface Charge, and Entrapment Efficiency of Lipid and Hybrid Protein–Lipid Nanocarriers Loaded with Sambucus nigra

**Figure 1:**
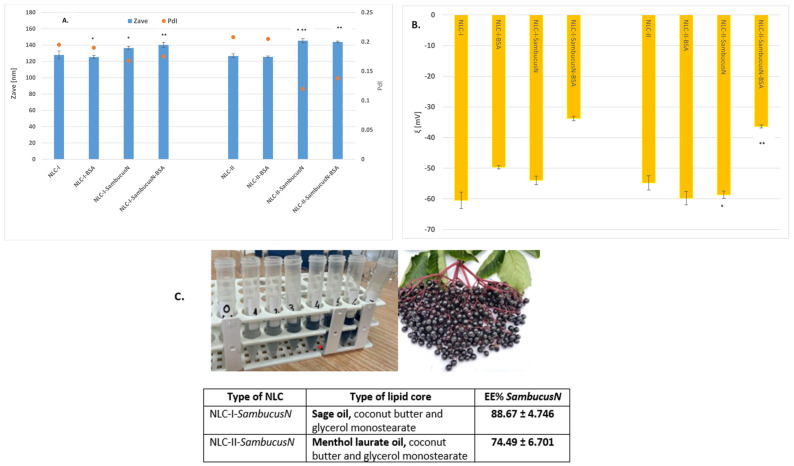

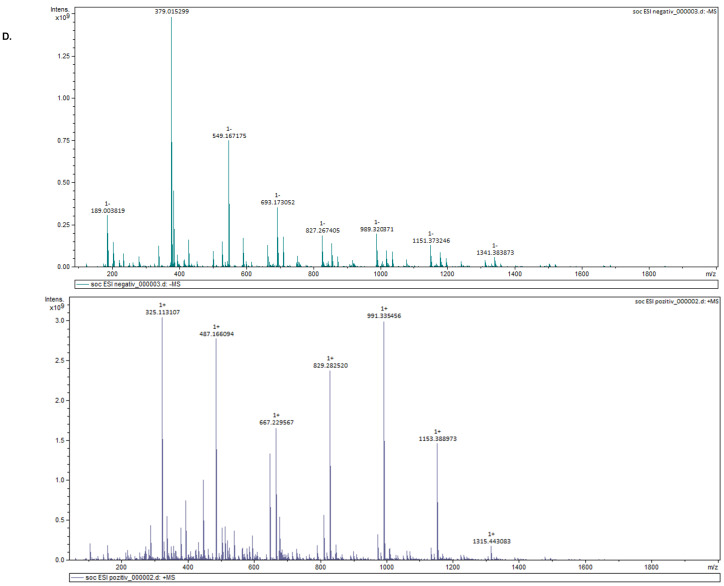
Different quality attributes of lipid and hybrid BSA−lipid nanocarriers hosting *SambucusN* extract: main diameter size (**A**), electrokinetic potential (**B**), entrapment efficiency (**C**), and FT-ICR MS/MS of *SambucusN* (**D**). All experiments were performed in triplicate. * *p* < 0.05; ** *p* < 0.005; *** *p* < 0.0005. Data are expressed as mean ± SD, *n* = 3 NLCI/II vs. other groups.

The construction of lipid nanocarriers and hybrid protein–lipid nanocarriers (bovine serum albumin-decorated NLC) was thought to host two categories of different lipophilic matrices, one consisting of the natural lipids MSG, coconut butter, and sage oil (NLC-I series), and another consisting of synthetic oil (menthyl laurate) associated with coconut butter and glycerol monostearate (NLC-II series). The stabilisation of these lipid cores was achieved with a mixture of cationic and amphiphilic surfactants (sodium cholate and phosphatidyl choline) to establish non-covalent attachment by means of weak interactions, such as electrostatic attractions and hydrogen bonds between the functional groups of surfactants and those of the polypeptide chain of BSA. The conventional NLC and those decorated with BSA protein and loaded with *Sambucus nigra* extract were prepared using a two-step procedure rationalised to preserve elderberry anthocyanins in the outer layer of the NLC. The characterisation of the NLC-I and -II formulations, in terms of nanocarriers size, surface charge, as well as entrapment efficiency of the *SambucusN* extract (using the spectrophotometric Folin–Ciocâlteu method), is summarised in Figure 1. The free lipid nanocarriers (without *Sambucus nigra*) presented comparable values of the lipid population diameters, around 125 nm, regardless of the type of lipid matrix used in the preparation. The entrapment process of the phytochemical extract into NLC led to a slight increase in average diameters, the diameters increasing by approx. 10–20 nm. For example, the Zave determined for NLC-I-*SambucusN*-BSA was 140.2 ± 3.26 nm versus 125.3 ± 2.08 nm for NLC-I-BSA (Figure 1A). These results converge towards the idea of the capture of phytochemicals in the surfactant shell, which directly results in an increase in the thickness of the nanocarriers. On the other hand, the polydisperse index was lower than 0.22, reaching a minimum of 0.175 ± 0.003 for NLC-I-*SambucusN*-BSA and 0.120 ± 0.022 for NLC-II-*SambucusN*, respectively. These values of PdI highlight the presence of lipid populations with a relatively homogeneous and monodisperse distribution.

Concerning the zeta potential, all lipid and hybrid NLC-*SambucusN* exhibited strongly negative values (Figure 1B), which was expected due to the presence of sodium cholate and phosphatidylcholine. The prediction of coalescence or flocculation was assigned by the creation of a surface layer that imprinted electrokinetic zeta values more electronegative than −50 mV for conventional lipid nanocarriers, e.g., ζ = −54.0 ± 1.40 mV and −58.7 ± 1.19 mV for NLC-I- and NLC-II-*SambucusN*, respectively. Covering the NLC with BSA protein led to the annihilation of the surface charges of the NLC and implicitly to a drastic change of the electrokinetic potential values. For instance, for NLC-I- and -II-*SambucusN*-BSA, the zeta potentials were −33.8 ± 0.75 mV and −36.4 ± 0.47 mV, respectively.

To quantify the entrapment efficiency of *SambucusN* extract into NLC and in BSA-decorated-based NLC, the extract was subjected to high-resolution mass spectrometry analysis (FT-ICR MS, on both positive and negative ionisations) and the Folin–Ciocâlteu spectrophotometric method. The monoisotopic mass spectra determined using FT-ICR MS (Figure 1D) identified the main actives in *SambucusN*, i.e., phenolic acids and flavonoid derivatives. Their experimental fragments (Figure 1D), *m*/*z* cationic and anionic, for example, ions of protonated molecules (ESI+) or those obtained via negative ionisation (ESI−), are in accordance with the *m*/*z* masses reported in the literature [46,47,48,49,50,51,52]: 300.996 *m*/*z* ESI+ (ellagic acid, C_14_H_6_O_8_), 325.113 *m*/*z* ESI+ (p-coumaric acid-4-O-glucoside, C_15_H_18_O_8_), 355.102 *m*/*z* ESI+ and 353.087 *m*/*z* ESI− (5-caffenoylquinic acid, C_16_H_18_O_9_), 377.084 *m*/*z* ESI+ (chlorogenic acid, C_16_H_18_O_8_), 447.093 *m*/*z* ESI− (quercetin-3-glucoside, C_21_H_20_O_11_), 449.107 *m*/*z* ESI+ (quercetin-3-glucoside, C_21_H_20_O_11_), 463.088 *m*/*z* ESI− (kaempherol-3-glucoside, C_21_H_20_O_12_), 465.385 *m*/*z* (delphinidin-3-glucoside, C_21_H_21_O_12_), 495.113 *m*/*z* (epigallocatechin-7-O-glucuronide,C_22_H_24_O_13_), 549.167 *m*/*z* ESI− (quercetin-3-malonyl glucoside, C_24_H_22_O_15_), 595.165 *m*/*z* ESI+ (cyanidin-3-rutinoside, C_27_H_31_O_15_), 827.267 *m*/*z* ESI− (Naringin-6-malonate, C_36_H_44_O_22_), and 1153.388 *m*/*z* ESI+ (cinamtannin A2, C_60_H_50_O_24_). 

The polyphenolic content from *SambucusN* was determined by using the spectrophotometric Folin–Ciocâlteu method, according to ISO 14502-1:2005 [53]. The polyphenolic content in the extract, calculated and expressed as the gallic acid (GA) equivalent, was 2305 ± 1.2 mg of GAE/100 g of dry extract. The amount of phenolic content determined in our study is comparable to that reported by Młynarczyk et al. [25]. According to quantitative determination, the capture of the phytochemical mixture was more efficient in NLC-I-*SambucusN* prepared with a lipid blend containing sage oil (Figure 1C). The value of *SambucusN* entrapment efficiency for both kinds of nanocarriers was 88.67% ± 4.75 for NLC-I-*SambucusN* versus 74.49 ± 6.70 for NLC-II-*SambucusN* prepared with menthyl laurate. Most likely, the core of the NLC-I matrix allowed the accommodation of a relatively low amount of phytochemicals owing to the structural variability of the fatty acids from the triacylglycerols present in sage oil, despite their preferential affinity in the coating created by the surfactants. In the case of NLC-II, the rather rigid terpenoid structure of menthyl laurate did not allow the accommodation of *SambucusN* into the core, and as result, the phytochemicals from the extract were distributed exclusively in the outer shell.

### 2.2. FT-IR Spectroscopy of NLC-SambucusN-BSA

**Figure 2:**
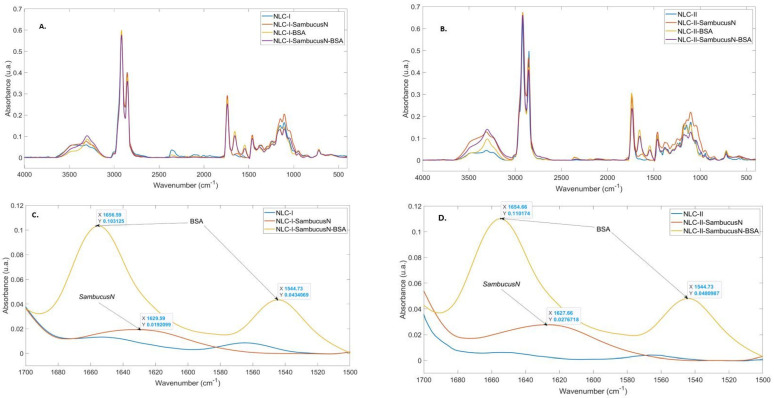
Comparative ATR-FTIR spectra of coated and uncoated NLC-I−*SambucusN* (**A**) and NLC-II−*SambucusN* (**B**). Details on ATR−FTIR spectra of NLC-I (**C**) and II−*SambucusN* (**D**) coated and uncoated with protein.

The comparative analysis of the NLC lyophilised via ATR-FTIR spectroscopy (Figure 2A,B) leads to spectra of similar general shape, exhibiting vibration bands characteristic of the structures of the glycerides, surfactants, and BSA involved in the synthesis; for example, the stretching of Csp3-H and Csp2-H bonds (3050–2760 cm^−1^) and C=O (ester groups) stretching at 1730–1740 cm^−1^. In addition, the spectra of the NLC containing BSA exhibit two distinct bands of amide I (1650 and 1655 cm^−1^, respectively) and amide II (1550 and 1540 cm^−1^, respectively). A very important feature of the amide I band is the shifting from the BSA (1643 cm^−1^) compared with BSA-decorated NLC-*SambucusN*, which can be attributed to changes in the secondary structure of the protein (an increase in the number of α-helix regions) due to the appearance of weak hydrogen bonds between BSA and the functional groups of the surfactants used for the stabilisation of the lipid core. This is also in agreement with the increase in the intensity of the O-H and N-H vibration modes at ~3300–3500 cm^−1^ and the slight redshift of the bands due to the appearance of hydrogen bonding. In addition, in the case of NLC-II-type carriers, this increase is more accentuated, indicating a stronger interaction than in the case of NLC-I. Considering that the difference between the formulations is the composition of the lipidic core, it can be inferred that the surfactant layer also interacts differently with the two types of lipid matrices.

The ATR-FTIR spectra of polymer BSA-coated NLC loaded with *SambucusN* extract are similar to the spectra of free NLC-BSA. To highlight the presence of polyphenols, the ATR-FTIR spectra of the coated and uncoated NLC-*SambucusN* were compared. Figure 2C, D exhibits an expanded domain of the ATR-FTIR spectra of coated and uncoated NLC-II-*SambucusN*. In the case of uncoated NLC-*SambucusN*, the band from ~1630 cm^−1^, which corresponds to the stretching of the C=C bonds from the aromatic rings of the polyphenols, confirms the presence of *SambucusN* in the surfactant layer of the nanocarrier (in accordance with the hydrophilic character of *SambucusN* extract). The disappearance of this band in the spectra of the BSA-coated sample can be explained by considering the synthesis method, the process through which the surface layer containing the polyphenols is covered completely with the BSA biopolymer.

### 2.3. Fluorescence Behaviour of Conventional and Hybrid NLC-SambucusN

**Figure 3:**
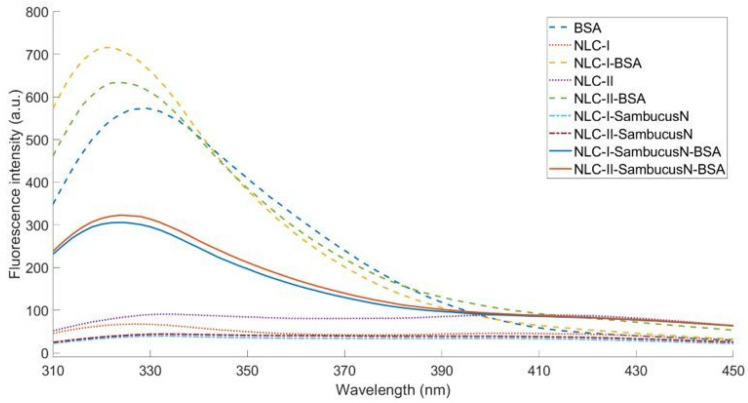
Comparative fluorescence spectra of the conventional NLC and hybrid BSA-decorated NLC, loaded or free of *SambucusN* extract.

Fluorescence spectroscopy is a method that can be used to study the interaction between proteins and ligands. Figure 3 exhibits the fluorescence spectra of all the NLC formulations described in this paper together with the fluorescence profile of pristine BSA. Having no fluorophores, the uncoated NLC solids do not present fluorescence. On the other hand, BSA yields a strong emission band at ~330 nm owing to the amino acid residues, mainly Trp134 and Trp213 [54]. The association between BSA and NLC leads to an enhancement in fluorescent emission. This phenomenon can be associated with the large specific surface area characteristic for nanosized carriers, allowing for more interaction centres and hence giving rise to a more intense fluorescence effect [55].

The loading of the coated NLC leads to a decrease in fluorescence, a process called quenching. This occurs as a result of environmental changes affecting the fluorophores, with each formulation showing up to a 3-fold decrease in intensity due to the presence of *SambucusN* extract. These results align with those of Yu et al. [56], who studied the interactions of BSA with tea polyphenols and observed up to a 2-fold decrease in the fluorescence properties of the protein upon binding of polyphenols due to the formation of hydrogen bonds between the hydroxyl group of the polyphenols and the amino acid residues of BSA. Additionally, changes in BSA conformation are evidenced by the blue shift in the emission peak (329 nm for native BSA vs. 322–324 nm for BSA-coated NLC). Das et al. [57] attributed this shift to the positioning of *Trp* residues in more hydrophobic pockets upon binding to nanoparticles.

### 2.4. In Vitro Assessment of Antioxidant Activity

**Figure 4:**
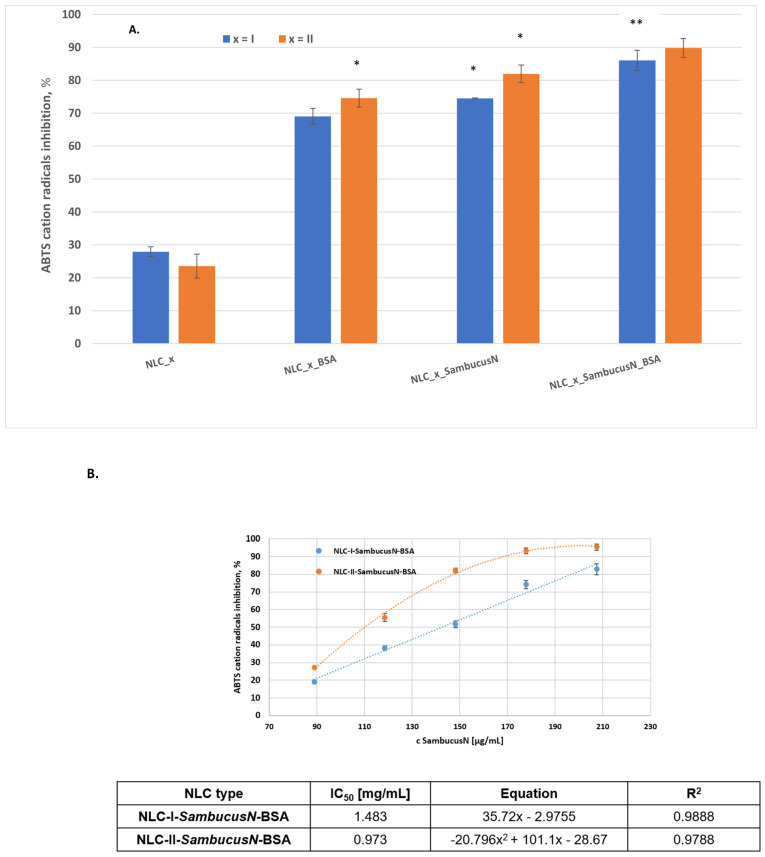
Antioxidant activity of NLC formulations: scavenging activity of ABTS radical cations by NLC-I and -II *SambucusN* with and without BSA polymer (**A**); dose−response curves and IC50 values of BSA-coated NLC (**B**). All experiments were performed in triplicate. * *p* < 0.05; ** *p* < 0.005.

Oxidative stress is recognised as a key factor in triggering drug resistance in chemotherapy protocols, primarily through the activation of HIFα and the overexpression of Nrf2 as a pivotal transcription factor [58]. The phenolic compounds in *Sambucus nigra* extract, including both flavonoids and phenolic acids, are known to be potent antioxidants. However, they unfortunately exhibit an inherent instability and compromised bioavailability [59]. TEAC and IC50 results (Figure 4) revealed that NLC-I and -II manifested ABTS cation radical-scavenging properties, mainly due to their hydrogen-donating ability, though to varying degrees. TEAC values ranged from 167 to 212.74 mM of Trolox/g of extract [60]. As shown in Figure 4A, the scavenging activity of long-life cationic radicals by NLC-I/II loaded with *SambucusN* was significantly higher than that of free NLC. The improved antioxidant properties of NLC are attributed to the presence of hydroxyl and carbonyl groups in the structure of polyphenols and the presence of conjugated or separated double bonds, which enhance their ability to annihilate ABTS cationic radicals. These results could be attributed to the larger specific area of the nanocarriers for the chemical quenching of the radicals present in the system. In other studies, it was observed that *SambucusN* encapsulated in nanoparticles exhibited higher antioxidant and radical-scavenging activities than the free extract solubilised in ethanol solutions [61].

Remarkably, the antioxidant activity of NLC-I and -II-*SambucusN* was significantly enhanced by the albumin biopolymer coating. Thus, the maximum annihilation power of ABTS cationic radicals determined for the two categories of NLC-hybrids was 89.81 ± 4.84% and 86.01 ± 3.07% for NLC-I and -II *SambucusN* covered with a BSA biopolymer, versus 74.47 ± 1.15% and 81.93 ± 2.70% determined for NLC-I and -II-*SambucusN* without BSA (Figure 4A). This behaviour can be attributed to the role of the BSA biopolymer, which improves the solubility of NLC formulations in the environment where the radicals are present (due to the amphiphilic BSA layer) and with a potential antioxidant synergy between BSA and polyphenols from extract. Similarly, the encapsulation of other natural compounds, such as beta-carotene or quercetin in protein-based nanoparticles, has been shown to significantly improve DPPH radical-scavenging activity [62]. It is worth noting that NLCs without *SambucusN* extract exhibited a moderate scavenging activity (less than 30%), and therefore, the lipid excipients of the carrier had a minor influence on the antioxidant action of NLC-I and -II with the phytochemical extract. Taken together, these results conclude that NLC-I/II *SambucusN* BSA had the highest antioxidant potential. The ability of NLC to reduce ABTS cationic radicals was also investigated using the IC50 value. The IC50 is defined as the concentration of the tested formulation needed for scavenging ABTS radicals in the solution by 50%. The lower the IC50 value, the stronger the antioxidant activity of the NLC. The relationship between TEAC and IC50 values of NLCs showed a good fit to a linear model in the case of NLC-I-*SambucusN*-BSA (R^2^ = 0.9888) and to a second-degree polynomial model for NLC-II-*SambucusN*-BSA (R^2^ = 0.9788), as shown in Figure 4, indicating slightly different trends in ABTS radical-scavenging activities. At higher concentrations, the antioxidant properties decrease due to some steric accessibility and ABTS assay limitations. Most likely, the high concentration of BSA-decorated NLC-*SambucusN* influences the change in linearity in a polynomial function. At high antioxidant concentrations and implicitly, the abundance of hydroxyl groups leads to the appearance of steric-hindrance effects. This aspect is also supported by the results obtained by Schaich et al., who showed that critical effects of steric accessibility resulted in a significant influence of the antioxidant concentration on the reaction rate [63]. Small phytochemicals reduce stoichiometric ABTS^●+^ per -OH phenolic, and the reaction response remains linear as the antioxidant concentration increases. However, the reaction becomes increasingly hindered at higher antioxidant concentrations as the structural complexity of the bioactive-derived *SambucusN* increases. This happens because the secondary rings in the polyphenols interfere with the access of the hydroxyl groups to ABTS^●+^; in other words, steric hindrance occurs. 

The IC50 values for the two representative NLCs, extrapolated from Figure 4B, were 1.483 mg/mL and 0.973 mg/mL, which reveals the superior antioxidant activity of NLC-II-*SambucusN*-BSA, which includes menthyl laurate in its composition; these might be due to the better capturing of some lipophilic flavonoids from *SambucusN* into the lipid blends with menthyl laurate. It should be mentioned that the determined IC50 values considered the entire lipid nanocarrier formulation, considering the potential involvement of the composition of the two oils of sage and menthol in combating dangerous reactive species. These results are in agreement with data recently reported by Rohmah et al. [64].

### 2.5. In Vitro Antitumour Potential of NLC-I and II-SambucusN-BSA

Understanding the mechanisms responsible for the formation and progression of tumours is crucial for developing effective cancer treatments. One of the key factors is the amplification of the cell-division process, which allows tumour cells to proliferate uncontrollably [65,66]. Another important factor is the inhibition of the apoptotic process, which is a natural mechanism of programmed cell death that eliminates damaged cells. In tumour cells, this process is inhibited, allowing them to survive and continue dividing, even when they are abnormal or damaged. By targeting these mechanisms, cancer treatments can prevent the growth and spread of tumours [67]. By analysing the DNA content, cell cycle analysis by flow cytometry can establish the percentage of each phase of the cell cycle (G0/G1, S, and G2/M), providing a complete understanding of the NLC action mechanisms [68,69]. Additionally, the apoptotic process can also be determined through flow cytometry using double labelling with Annexin V-FITC/PI to enable the differentiation between live cells and the cells undergoing apoptosis or necrosis. By co-opting the two categories of active principles (sage oil/menthyl laurate with *SambucusN* extract) into NLC and BSA-covered NLC, we pursued the hypothesis of their antitumour action using cell lines derived from human adenocarcinomas of the colon (LoVo), breast (MCF-7), and ovary (SKOV-3), compared with normal endothelial cells from the human umbilical vein (HUVEC) used as a control. 

The predicted antitumour activity is sustained by the extensive scientific research on the chemoprevention and therapeutic potential of *SambucusN* extract and their major phytoconstituents. Some bioactive-derived *SambucusN* may provide a scaffold that could target specific signalling pathways to exhibit their bioactivities. In this regard, *Sambucus*-derived anthocyanins and other phenolics were recently demonstrated to interact with the tumour lipid membrane [43]. *SambucusN*-derived bioactives increased the packing order in the tumour lipid membranes, inhibited the activity of COX-1 and COX-2, and mediated their cytotoxicity against MCF-7 breast cancer cells [70]. The lipid tumour membrane was also the primary target of elderberry bioactive soy lecithin liposomes [70]. According to Banach et al., *Sambucus N* extract inhibits both the initiation phase and the development phase of carcinogenesis, which leads to the significant cytotoxic properties that inhibit cancer cell proliferation [34]. Moreover, the structure–activity relationship of flavonoids, phenolic acids, anthocyanins, and tannins from *SambucusN* that may act synergistically is also an important factor. Periera et al. indicated that flavonoids from *SambucusN* are responsible for the cytotoxic effect on human bladder carcinoma T24 cells [35]. In vivo studies of kaempferol on pancreatic cancer cells concluded that it could induce apoptosis via the regulation of ROS levels [37]. Kaempferol’s mechanism on MDA-MB-231 and BT-474 breast cancer cells was mainly assigned to inhibiting tumour cell growth and inducing their apoptosis [71]. The potential antitumour mechanism exerted by quercetin involves many pathways, such as the necroptosis of breast MCF-7 cancer cells [72], induction of ovarian tumour cell apoptosis via an interplay between quercetin and miRNA [39], or modulating the ROS, Akt, and NF-κB pathways for efficacy against prostate cancer [40]. Chlorogenic acid also showed cytotoxicity against cancer cells; it inhibited the tumour growth and invasion of cancer cells by targeting p53 and reactive oxygen species [73]. Cyanidin-3-glucoside induces the apoptosis of breast cancer cells, inhibits melanoma cell proliferation [m], and weakens angiogenesis by inhibiting VEGF [74]. Anthocyanin-enriched extracts of elderberries were found to show cytotoxicity against human A2780 ovarian, MCF-7 breast, and HCT116 colon cancer cells [34,44] via the regulation of MAPK, AMPK, and NF-kB signalling pathways.

**Figure 5:**
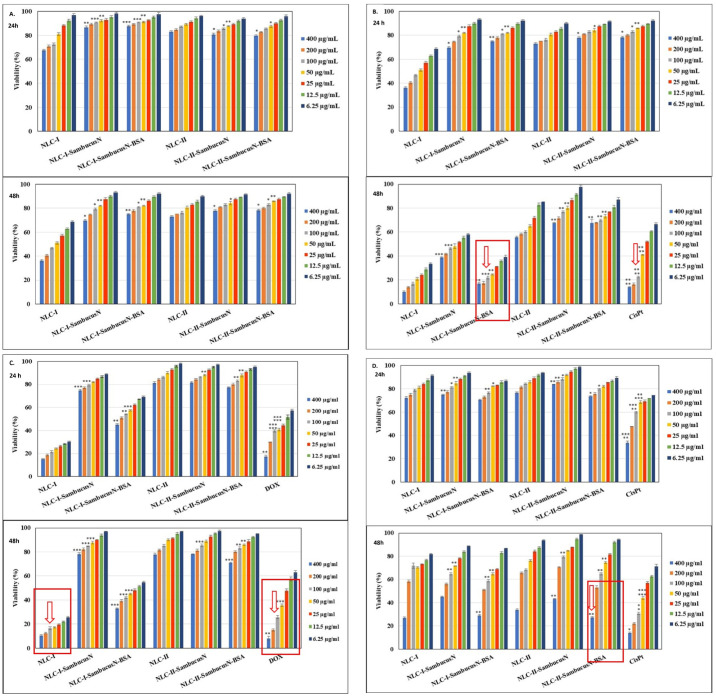
Effect of the conventional NLC-I and -II and hybrid BSA-coated NLC-I and -II, loaded with *SambucusN* extract, on the cell viability of the following: (**A**) normal HUVEC cells; (**B**) LoVo colon cancer cells; (**C**) MCF-7 breast cancer cells; and (**D**) SKOV-3 ovary cancer cells after 24 h and 48 h of incubation with developed NLC and chemotherapy drugs, cisplatin and doxorubicin. All experiments were performed in triplicate. * *p* < 0.05; ** *p* < 0.005; *** *p* < 0.0005. Data are expressed as mean ± SD, *n* = 3 NLCI/II vs. other groups.

**Figure 6:**
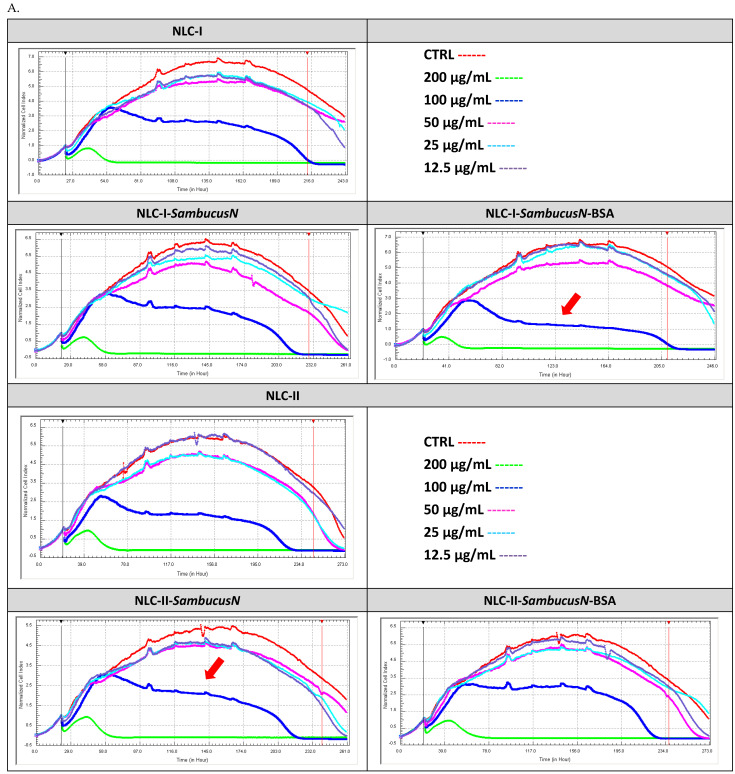

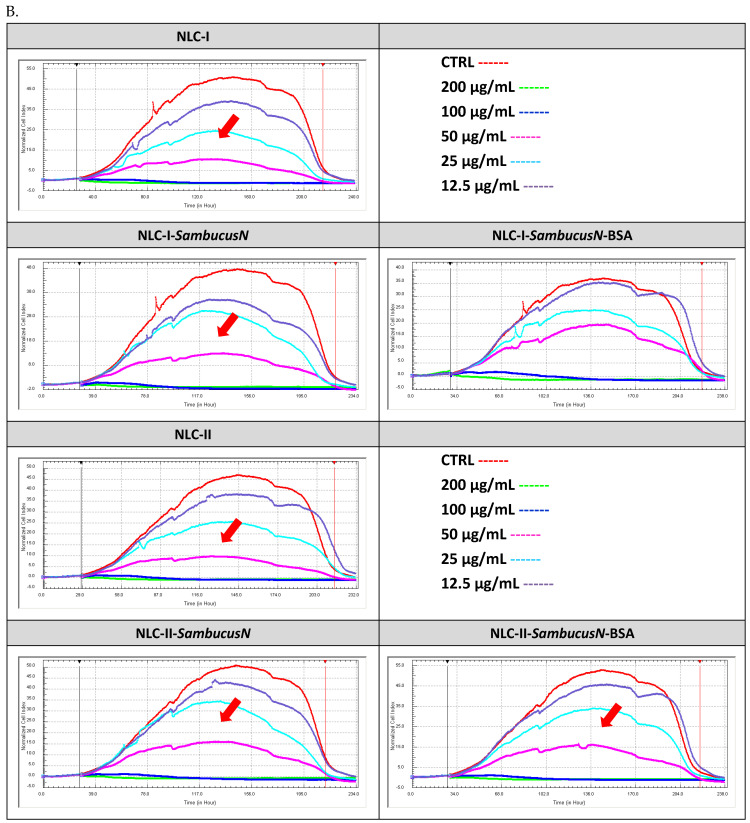

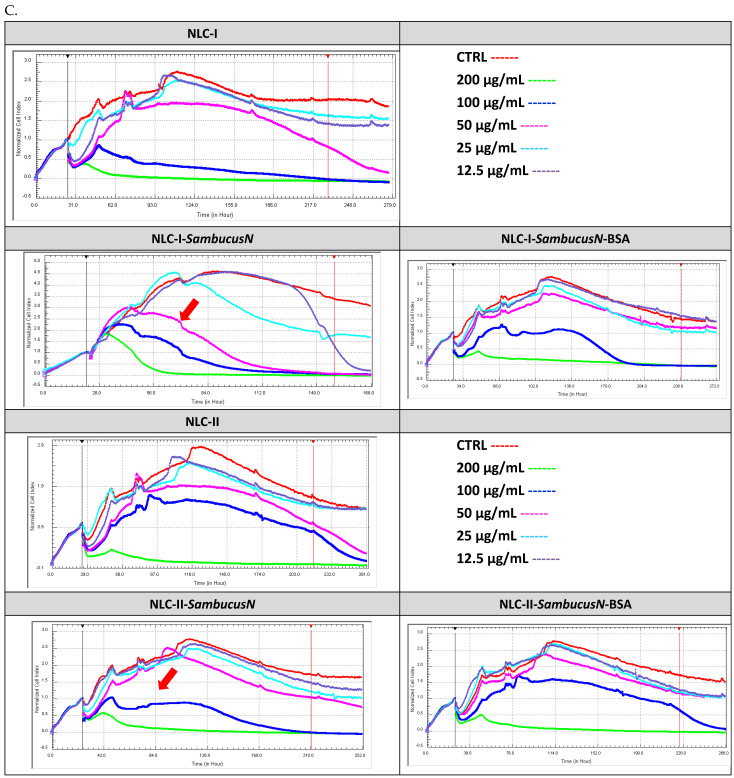

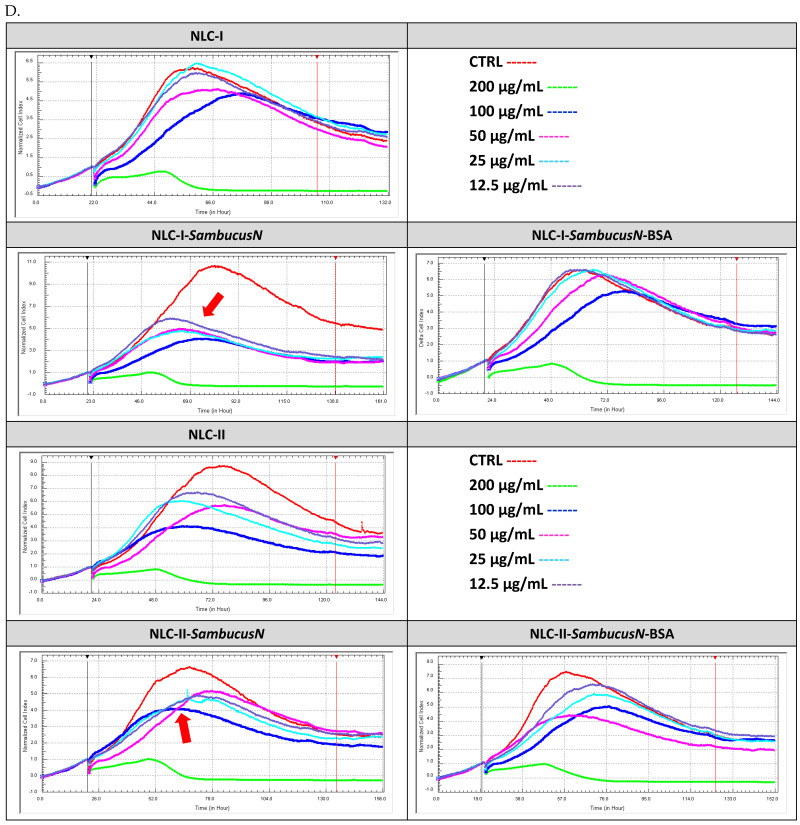
Real-time cell analysis (RTCA) analysis recorded on normal HUVEC cells (**A**), normal LoVo colon cancer cells (**B**), normal MCF-7 breast cancer cells (**C**), and normal SKOV-3 ovarian cancer cells (**D**) for conventional NLC-I and -II and hybrid BSA-coated NLC-I and -II, with and without *SambucusN* extract.

**Figure 7:**
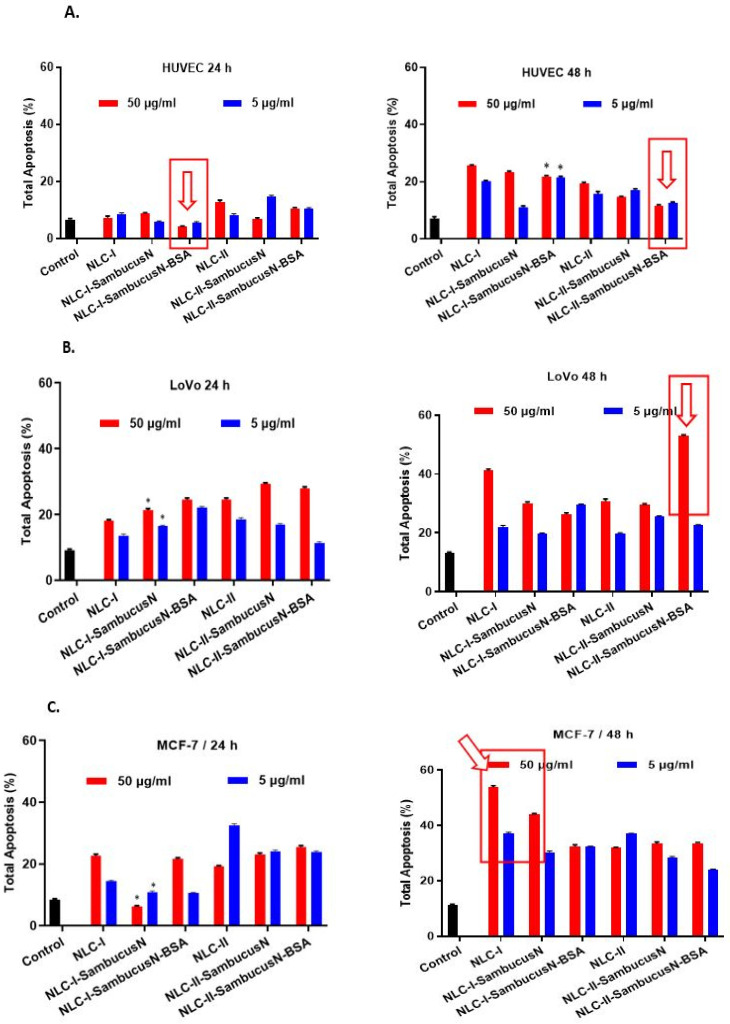

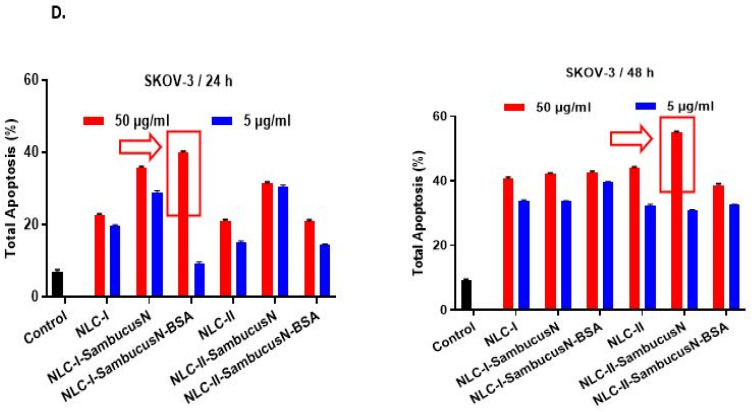
Apoptotic process induced by various categories of NLC on different cancer cells: (**A**) normal HUVEC cells; (**B**) LoVo colon tumour cells; (**C**) MCF-7 breast tumour cells; (**D**) SKOV-3 ovary tumour cells. All experiments were performed in triplicate. * *p* < 0.05. Data are expressed as mean ± SD, *n* = 3 NLCI/II vs. other groups.

**Figure 8:**
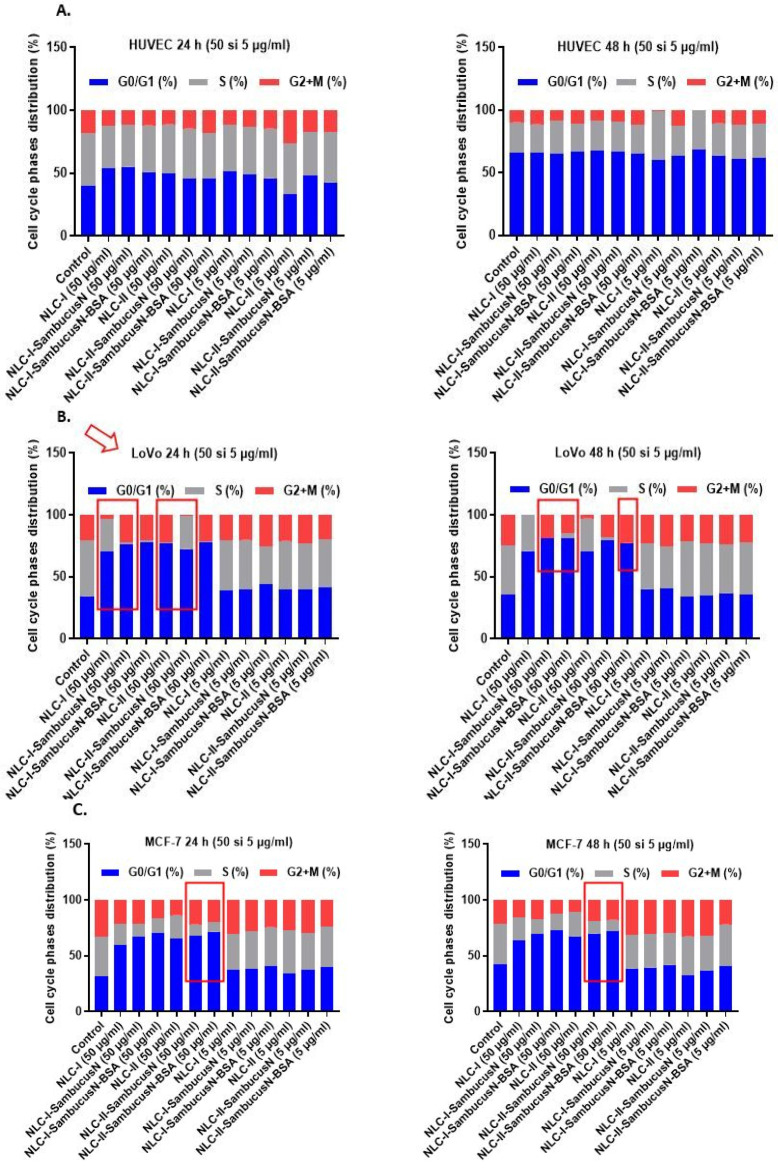

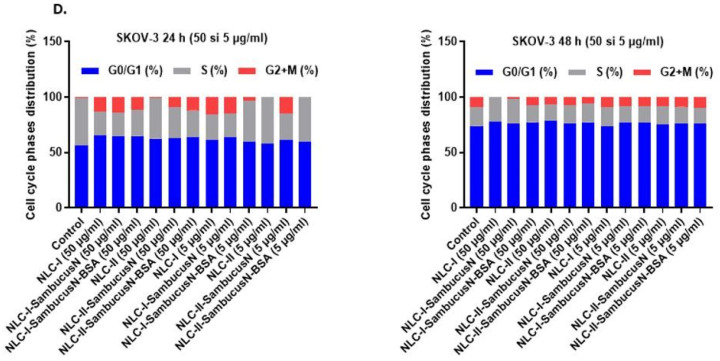
The influence of the conventional NLC and hybrid BSA-coated NLC on cell cycle in tumour cells: (**A**) normal HUVEC cells; (**B**) LoVo colon tumour cells; (**C**) MCF-7 breast tumour cells; (**D**) SKOV-3 ovary tumour cells.

In this study, analysing both the apoptotic process and cell cycle progression provides a comprehensive understanding of the effects of the conventional and hybrid-NLC-*SambucusN* on the tumour cells. The dose–effect curves were generated for each cell line (normal and tumoural cells) to identify the optimal concentration of each albumin-decorated NLC-*SambucusN* that would provide the inhibition of biological responses (Figure 5, Figure 6, Figure 7 and Figure 8). Untreated cells were considered negative controls (control), and cells treated with cisplatin (Cis-Pt) and doxorubicin (DOX) were positive controls (these substances being used in cancer treatment as cytostatic agents). Concentration-dependent reductions in cell viability count were observed in polyphenol-rich *SambucusN*-loaded nanostructured lipid carriers and polymer–lipid hybrid nanocarriers. They influence proliferation and different complex processes, such as the apoptotic process or the cell cycle of tumour cells. NLC I and NLC II in all formats inhibited the proliferation of tumour cells depending on the concentration and treatment period (24 or 48 h), acting on the cell cycle differently depending on the type of tumour cells.

#### 2.5.1. Cell Cytotoxicity of the Conventional and Hybrid BSA-Decorated NLC-S*ambucusN*

To establish the effect of the developed conventional and hybrid NLC on tumour cell viability, different concentrations of conventional NLC-I and -II and BSA-coated NLC-I and -II, loaded with S*ambucusN* extract, ranging from 6.25 mg/mL to 400 mg/mL, were tested after 24 and 48 h of incubation. Equivalent concentrations of blank NLCs were also tested as a control. For the concentrations between 6.25 and 100 mg/mL, the NLC-I and -II systems with *SambucusN* did not show any toxic effect on normal endothelial HUVEC cells (Figure 5A), with viability values between 85% and 98% (treatment for 24 h). At 48 h, the viability was slightly lower, but most normal cells remained viable, except for blank NLC-I. According to Jablonská E et al., cell viability values > 70% are considered “nontoxic” [75]. These results confirm the biocompatibility of NLCs, a finding that is in line with other studies that have demonstrated good tolerability of NLCs in HUVEC epithelial cell lines. Figure 5B–D indicate the % cell viability at different concentrations in three categories of tumour cell lines: LoVo, MCF-7, and SKOV-3. At all concentrations, blank NLC-I displayed greater cytotoxicity on LoVo colon tumour cells (Figure 5B) and MCF-7 breast tumour cells (Figure 5C) compared to conventional chemotherapy DOX and Cis-Pt drugs. It is noteworthy that for a concentration of 100 μg/mL and 48 h, NLC-I-*SambucusN*-BSA behaves similarly to the Cis-Pt drug, leading to the cell viability of LoVo colon tumour cells of 21.81 ± 1.18% (Figure 5B). NLC-II, in any form, with or without loading of phytochemical extract, does not show a notable inhibition of the proliferation of LoVo colon tumour cells.

Regarding the behaviour of MCF-7 breast tumour cells, NLC-I with sage oil content assured an advanced toxic effect, e.g., 10.32 ± 0.97%, for 48 h treatment. Comparatively, 100 μg/mL of NLC-I leads to 16.47 ± 1.16% viability versus 25.72 ± 1.35% viability after treatment with synthetic DOX (Figure 5C). This result confirms a notable contribution to inhibiting cancer proliferation and could be explained by the role of sage oil. Sage oil is known for its ability to manifest antitumour action in vitro and in vivo [76]. The efficiency of NLC against the proliferation of SKOV ovarian cancer cells is quite low (Figure 5D), with a maximum of cell inhibition occurring only for NLC-II-*SambucusN*-BSA after a 48 h treatment (e.g., ~27% for a concentration of 400 mg/mL).

#### 2.5.2. Real-Time Cell Analysis of Proliferation of Tumour Cells Treated with Lipid and Hybrid Nanocarriers

The cytotoxic action vs. proliferative effect induced by developed NLC on various tumour cell lines was studied using an RTCA assay. The proliferation curves recorded on an extended period, 0 to 243 h, were performed after LoVo, MCF-7 and SKOV-3 tumour cells treatment with NLC concentrations ranging from 12.5 to 200 μg/mL; normalisation was achieved using the untreated cell proliferation curve (red curve in Figure 6). The effect of NLC on normal HUVEC cells reveals that concentrations of 12.5, 25, 50 μg/mL do not significantly affect the proliferation of these endothelial cells. Concentrations higher than 100 μg/mL NLC appear to influence normal cell proliferation, especially after 80 h of treatment (Figure 6A).

Through a comparative evaluation of the antitumour efficacy of NLC on the three types of tumour lines, it was observed that the most pronounced antitumour action was manifested in the LoVo colon cell line. For instance, an advanced inhibition activity was observed at concentrations of 50 μg/mL of NLC-*SambucusN* (the effect indicated by the arrow in Figure 6B). Concentrations of 100 μg/mL of NLC-*SambucusN* led to a plateau, corresponding to a cell death activity. Thus, LoVo tumour cells are sensitive to all NLC analysed, with and without BSA covering, when used in concentrations higher than 50 μg/mL. Among the analysed NLC, NLC-I-*SambucusN*-BSA showed a clearly superior ability to inhibit the proliferation of LoVo tumour cells.

Following the treatment of MCF-7 breast tumour cells, it is observed that NLC, in all forms, used at a concentration of 200 µg/mL, significantly inhibits tumour proliferation. A total of 100 µg/mL of NLC-I-*SambucusN* with and without BSA is more effective in inhibiting the proliferation of MCF-7 tumour cells if the duration of the treatment extends beyond 48 h (Figure 6C). Regarding NLC-II, the proliferation tumour activity was more inhibited by NLC-II-*SambucusN* compared to the NLC covered by BSA protein (Figure 6C). In conclusion, the proliferation activity was more affected by NLC-I and -II-S*ambucusN*-BSA compared to without BSA (as indicated by the red arrow, Figure 6C).

A relatively similar trend in tumour-proliferation inhibition in the analysed NLCs was detected for SKOV-3 ovarian tumour cells. NLC-I-*SambucusN* prepared with sage oil was the most efficient at all concentrations tested (Figure 6D). In contrast, the BSA coating of the nanocarriers did not lead to an improvement in the antitumour proliferation action.

Regarding the NLC-II containing menthyl laurate, NLC-II-*SambucusN* and NLC-II-*SambucusN*-BSA significantly inhibit the proliferation of SKOV-3 tumour cells for a concentration of 100 µg/mL and an extended treatment over 48 h (Figure 6D).

#### 2.5.3. The Effect of the Hybrid Albumin-Decorated NLC-*SambucusN* on the Apoptosis Process

The results of the previous analyses led to the selection of working concentrations of 5 μg/mL and 50 μg/mL NLC to follow the effects on the apoptotic process, quantified using flow cytometry. To analyse the toxic action of these compounds on normal cells, NLC was applied under the same conditions on normal HUVEC cells. The data show that NLC-I-*SambucusN*-BSA (24 h) and NLC-II-*SambucusN*-BSA (48 h) protect normal cells from entering apoptosis compared to the effect induced by NLC-I (24 h) and NLC-II (24 h) regardless of their action time (Figure 7A).

The apoptotic process in LoVo untreated tumour cells is very low for 24 h of treatment. Although the effect induced by NLC-I and II-*SambucusN* with and without BSA on LoVo tumour cells after 24 h treatment did not exceed 18% apoptosis, the treatment of LoVo tumour cells for 24 or 48 h with the three forms of NLC-II caused an increase in the apoptotic process that was 2–4 times higher compared to untreated cells (control). An amplified apoptotic process was found for 50 μg/mL of NLC-II-*SambucusN*-BSA, which induced over 50% apoptosis after 48 h of treatment (Figure 7B), which was four times higher compared to the control group.

Regardless of the concentration used, all types of NLC-II seem more effective regarding MCF-7 tumour cell apoptosis than the effect induced by the NLC-I series, especially for 24 h of treatment. NLC-II-*SambucusN* with/without BSA induced a slight concentration-dependent increase in apoptosis of MCF-7 breast tumour cells after 24 h. However, a prolonged treatment (48 h) with NLC-I and NLC-I-*SambucusN* appears to be more efficient regarding the apoptosis of MCF-7 cells, e.g., the apoptotic process is amplified by 5× (50 μg/mL) with NLC-I compared to the control (Figure 7C). 

The results determined for SKOV-3 ovarian cancer cells showed that NLC-I, NLC-I-*SambucusN,* and NLC-I-*SambucusN*-BSA at 50 μg/mL activate the apoptosis process of tumour ovarian cells both at 24 and 48 h. NLC-II acts in a similar manner, amplifying the apoptotic process of tumour cells compared to the control. For instance, NLC-II-*SambucusN* at 24 h amplifies the apoptotic process by 4× regardless of the concentration compared with the untreated cells (Figure 7D). By far, the most advanced capacity to produce apoptosis was detected for NLC-II-*SambucusN* at 48 h, the total apoptosis being ~57% (Figure 7B).

#### 2.5.4. Influence of NLC-*SambucusN* and NLC-*SambucusN*-BSA on the Cell Cycles of Normal and Tumour Cells

The results obtained via flow cytometry analysis indicate that the treatment of lipid and hybrid protein–lipid nanocarriers with and without *SambucusN* extract does not significantly influence the cell cycle phases in normal HUVEC cells (Figure 8A). 

In contrast, untreated LoVo tumour cells show a higher S phase, which is indicative of intense proliferation activity. When NLC-I, NLC-I-*SambucusN*, and NLC-I-*SambucusN*-BSA are applied in 50 μg/mL concentrations, they induce cell cycle arrest in LoVo tumour cells in the G1 phase. This is accompanied by a significant decrease in the S phase, and this effect persists even when the treatment duration is extended to 48 h. For example, the best results in terms of total cessation of proliferation activity and advanced induction of cell cycle arrest were determined for treatment with 50 mg/mL of NLC-I-*SambucusN* and NLC-II-*SambucusN*-BSA for 48 h (Figure 8B).

Likewise, the treatment of MCF-7 tumour cells with 50 μg/mL of NLC-I and NLC-II effectively blocks the cell cycle in the G1 phase and induces a dramatic decrease in the S phase. NLC-II-*SambucusN* and NLC-II-*SambucusN*-BSA demonstrate greater efficacy than the type I nanocarrier series prepared with sage oil both at 24 h and 48 h (Figure 8C).

The cell cycle of untreated SKOV-3 tumour cells shows that these cells have a large S phase that can be associated with an intense proliferation process. All types of lipids and hybrid protein–lipid nanocarriers act in the sense of decreasing the S phase of SKOV-3 cells accompanied by an increase in the G2 phase. These results emphasise that NLC-I and -II could inhibit the proliferation of SKOV-3 tumour cells by blocking the cell cycle in the G2 phase. Treatment of SKOV-3 cells for 48 h with three forms of NLC-I and NLC-II does not significantly modify the phases of the cell cycle distribution (Figure 8D).

In this research, the understanding of the antitumour action mechanisms of the albumin-decorated NLC-*SambucusN* mainly derives by inhibiting the proliferation of tumour cells, preventing them from achieving intense radical-scavenging oxygen activity by inducing apoptosis with an interplay between phytoconstituent-derived *SambucusN* and DNA content (cell cycle analysis) and by modulating the cell cycle phase (G0/G1, S, and G2/M), with a clear differentiation between live cells and the cells undergoing apoptosis or necrosis. In addition, a valuable role is attributed to albumin-targeting ability. Albumin efficiently contributes to the targeting ability of tumour cells because it binds to glycoproteins overexpressed in cancer cells, e.g., the gp60 receptor and SPARC [5,6], thus assuring the enhanced accumulation of albumin-decorated NLC-*SambucusN* inside the tumour cells. And last but not least, a nanosized effect enhanced the bioavailability of the *SambucusN* extract. The induction of necrosis, autophagy, and DNA lesions via silver nanoparticles capped with elderberry extract was reported by Filip et al. [77]. The biological activities involved proapoptotic proteins and tumour-suppressor protein p53. Anthocyanin-gold nanoparticles exhibited growth-inhibitory activity against human prostate cancer cells [78]. 

Medicine nanoparticle application has a low approval rate (less than 10%) due to quality, safety, and efficacy issues in preclinical and clinical trials [79]. Few albumin-associated pharmaceutical preparations are currently on the market; for instance, FDA and EMA have approved the first albumin-based delivery system, i.e., albumin nanoparticles with Paclitaxel (Abraxane) for various types of cancer, including breast cancer, non-small-cell lung carcinoma, and pancreatic adenocarcinoma [80]. There is ongoing and consistent research on the advancement of albumin-based nanocarriers to treat cancer. Because albumin assures a better absorption of nanoparticles in the tumour, mainly owing to the fact that it binds to SPARC protein, thus preventing the efflux mechanisms of the drug, researchers are exploring alternative lipid nanodelivery systems. Even though nanostructured lipid carriers/NLC have a strong scientific basis for an appropriate role in chemoprevention and may have alternative therapeutic potential in cancer disease therapy, no NLC has been approved yet for tumour therapy.

## 3. Materials and Methods

### 3.1. Materials

Phosphatidylcholine (PC), Tween 20 (TW 20), bovine serum albumin (BSA), Trolox (6-hydroxy-2,5,7,8-tetramethylchroman-2-carboxylic acid), potassium persulfate, 2,2azinobis-(3-ethylbenzthiazoline-6-sulfonic acid) (ABTS), gallic acid, anhydrous sodium carbonate, NaCl, and Folin–Ciocâlteu reagent were purchased from Sigma-Aldrich Chemie GmbH (Munich, Germany). Sodium cholate (NaCh) was purchased from Merck (Darmstadt, Germany). Glycerol monostearate (GMS) was acquired from Cognis GmbH (Monheim am Rhein, Germany). Sage oil (SO) and coconut butter (CB) were provided by SC Esprovita SRL (Arad, Romania) and SC Green Sens Distribution (Bucharest, Romania). The phytochemical mixture, *Sambucus nigra* extract, was supplied by Organic Herb Inc. (Changsha, China). Menthyl laurate (MnL) was synthesised according to Ion et al. by using a Steglich reaction, the method being previously reported [81]. Doxorubicin (DOX), cisplatin (Cis-diammineplatinum(II) dichloride, Cis-Pt), PBS/1 mM of EDTA, L-glutamine (Glu), penicillin (100 units/mL), Streptomycin (100 μg/mL), Dulbecco’s modified Eagle’s medium (DMEM), fetal bovine serum (FBS), Propidium Iodide (PI) (stock solution of 4 mg/mL of PI in PBS) and RNase A (stock solution of 10 mg/mL of RNase A) were purchased from Sigma-Aldrich (St. Louis, MO, USA). Annexin V-FITC kit and CycleTEST PLUS DNA Reagent kit were purchased from Becton Dickinson Biosciences, San Jose, CA 95131, USA. The stock solutions were prepared by dissolving the compounds in a minimum amount of DMSO and kept at −20 °C. The working solutions were prepared from the stocks with the culture medium before each experiment.

### 3.2. Preparation of Conventional and Hybrid BSA-Decorated Nanostructured Lipid Carriers

Modified high-pressure homogenisation (HPH), as previously reported by the authors [82,83], was used to prepare the conventional-NLC and albumin-decorated lipid nanocarriers loaded with *Sambucus nigra* extract. In outline, the melted lipid phase (consisting of GMS, CB, and SO) was combined with the aqueous phase containing the mixture of NaCh, TW 20, and PC surfactants and the *SambucusN* extract. The resulting pre-emulsions were kept for 15 min at 70 °C under stirring. The concentration of the total lipid phase was maintained at 10%. A total of 40 mL of dispersion and 40 mL of BSA aqueous solution at 10 mg/mL were combined under high-shear homogenisation for a minute at 9000 rpm and approximately 40 °C. This resulted in the BSA-coated NLCs. Using a Martin Christ Alpha 1-2 LD Freeze Drying System (Martin Christ, Osterode am Harz, Germany), aqueous dispersions of NLCs were lyophilised, and solid hybrid–NLC formulations were obtained without the use of a cryoprotective agent (0.05 mbar, −55 °C, 54 h).

### 3.3. Cell Cultures and Treatments

LoVo (human colon adenocarcinoma), MCF-7 (human breast adenocarcinoma), and SKOV-3 (human ovarian adenocarcinoma) human cancer cell lines were purchased from American Type Culture Collection (ATCC). A normal cell line, human umbilical vein endothelial cells (HUVEC), was used as a reference. Adherent cells were routinely maintained in culture in DMEM: F12 medium added using 2 mM of L-glutamine, 10% fetal bovine serum, 100 units/mL of penicillin, and 100 μg/mL of streptomycin (Sigma Aldrich, St. Louis, MO, USA) and incubated at 37 °C in 5% CO_2_ humidified atmosphere. After 24 h, when cells achieved around 60% confluence, they were treated with different concentrations of the compounds for different periods of time. Cis-Pt and DOX, conventional oncology drugs used in cancer treatments, can be used as a positive control of the experiments. Cell treatments of compounds Cis-Pt and DOX were carried out using concentrations of 400, 200, 100, 50, 25, 12.5, and 6.25 μg/mL of the drug. Then, cells from flasks were detached with a non-enzymatic solution of PBS/1 mM EDTA, washed twice in PBS, and used for proliferation/cytotoxicity assays or evaluation of apoptotic events using flow cytometry. Alternatively, cells were fixed in ice-cold ethanol/PBS (70:30) and kept until use at 4 °C for cell cycle analysis using flow-cytometry technique. In all experiments described in this study, all untreated cells were designated as control cells.

### 3.4. Characterisation Methods

#### 3.4.1. Characterisation of *Sambucus nigra* Extract

The high-resolution mass spectrometry analysis was carried out using a Fourier Transform–ion cyclotron resonance (FT-ICR) spectrometer, SolariX XR 15T (Bruker Daltonics, Bremen, Germany). The *Sambucus nigra* sample was introduced via direct infusion. Positive ESI ionisation was performed with a sample flow rate of 120 µL/h, with a nebulisation gas pressure (N_2_) of 2.2 bar at 180 °C and a flow rate of 3.5 L/min. Negative ESI ionisation was performed with a sample flow rate of 120 µL/h, with a nebulisation gas pressure (N_2_) of 2.8 bar at 180 °C and a flow rate of 3 L/min. The spectra were recorded over a mass range between 46 and 1200 amu at a source voltage of 3900 V.

#### 3.4.2. Particle Size and Polydispersity Index

The polydispersity index (PdI) and mean particle size (Z_ave_) of BSA-coated NLC and conventional NLC were measured using dynamic light scattering (DLS) with a Zetasizer Nano ZS (Malvern Instruments Ltd., Worcestershire, UK) at 25 °C and a 90° scattering angle. The lipid nanocarrier dispersions were diluted with water to obtain samples with an adequate scattering intensity. The intensity distribution was used to assess the particle size data. The average of three distinct measurements was used to calculate each Zave and PdI value.

#### 3.4.3. Zeta Potential Measurements

The electrical properties of the coated and uncoated NLC were measured using the electrophoretic light scattering method (Zetasizer Nano ZS, Malvern Instruments Inc., Worcestershire, UK). The zeta potential, ξ, was determined in a capillary cell using the Helmholtz–Smoluchowski equation (μe = ξε/η, where ε and η are the dielectric constant and solvent viscosity, respectively). Zeta potential (ξ) was obtained by converting the particle electrophoretic mobility (μe) measurement. The NLC dispersions (100 μL) were diluted with water (25 mL) prior to analysis, and a 0.9% NaCl solution was used to correct the conductivity to 50 μS/cm. Every measurement was made three times.

#### 3.4.4. Spectroscopic Characterisation (ATR-FTIR)

The ATR-FTIR spectra of solid NLC were recorded on a Bruker Vertex 70 Spectrometer (Bruker Optics GmbH, Ettlingen, Germany), equipped with a horizontal device for attenuated reflectance and diamond crystal on a spectral window ranging from 4000 to 400 cm^−1^.

#### 3.4.5. Fluorescence Assay

The fluorescent characteristics of the solid nanocarriers were obtained using an FP-650 Spectrofluorometer Jasco (Tokyo, Japan) equipped with a microcomputer for data recording. The samples were irradiated with excitation light with a wavelength of 285 nm, and the emission spectra were recorded.

#### 3.4.6. Entrapment Efficiency

Considering the hydrophilic character of the *Sambucus nigra* extract, the entrapment efficiency was determined by quantifying the quantity of polyphenols extracted in water from a known quantity of lyophilised NLC. The polyphenolic content, expressed as gallic acid equivalents (GAE), was determined by using the Folin–Ciocâlteu method, according to ISO 14502-1:2005 [53]. Therefore, over 0.15 g of uncoated NLC or extract, 1 mL of water was added, and the mixture was gently shaken, followed by the sampling of 0.5 mL of supernatant. The supernatant was mixed with 0.5 mL of 10% Folin–Ciocâlteu reagent (*v*/*v*) and 4 mL of 7.5% Na_2_CO_3_ solution. The mixture was allowed to react for 1 h at room temperature in a dark place, and then the absorbance of each sample was recorded at λ = 765 nm in triplicate by using a UV-Vis spectrophotometer V670 Jasco (Tokyo, Japan). A calibration curve (with R^2^ = 0.9919) was constructed by measuring the absorbance of gallic acid solutions with concentrations in the range of 0–100 mg/L. The entrapment efficiency of *SambucusN* was calculated using the following equation:

$$EE\left(\%\right)=\frac{C_{a}}{C_{e}}\times 100$$

where *C_a_* is the content of polyphenols in the analysed NLC, and *C_e_* is the content of polyphenols in the extract.

#### 3.4.7. ABTS-Scavenging Activity

The in vitro antioxidant activity was determined according to ABTS assay. Briefly, the cationic radical ABTS^●+^ was generated by the reaction between 7 mM of ABTS solution and 2.45 mM of potassium persulfate solution for 16 h in dark conditions and 4 °C. The ABTS^●+^ solution was normalised by adding ethanol to reach an absorbance of 0.700 (±0.01) at 734 nm (ABTS*). 

The samples were prepared by adding 3 mL of ABTS^*^ solution to 2 mL of NLC solution (5 mg/mL). The absorbance of the samples was measured after 4 min using ethanol as a reference, each sample being analysed in triplicate. The average of results was used for calculating the percentage of inhibition according to the following equation:

$$\%inhibition=\frac{A_{0}-A_{s}}{A_{0}}\times 100$$

where *A*_0_ is the absorbance of the blank (ABTS* and ethanol), and *A_s_* is the absorbance of the sample. 

The IC50 value was defined as the concentration at which 50% of ABTS^●+^ were scavenged and was determined by varying the NLC solution concentration in the range of 1 and 6 mg/mL.

#### 3.4.8. Real-Time Cell Analysis (RTCA)

To analyse the proliferation profiles of treated cells, we continuously monitored cell growth by using real-time cells analysis (RTCA) assay and an xCELLigence DP-System, ACEA Biosciences Inc., San Diego, CA, USA, which allows cell-based, label-free in vitro assays and real-time monitoring of cellular processes such as cell viability and cytotoxicity. Changes in a cell status, such as cell morphology, cell adhesion, or cell viability led to a change in cell index (CI), which is a quantitative measure of the cell number present in a well. Real-time impedance data were obtained and used to generate compound-specific profiles that are dependent on the biological mechanisms of action of each compound. Cells were cultured in DMEM culture medium with 2 mM of L-glutamine and 10% FCS and seeded in 100 μL of culture medium in 16 E-Plates cells (ACEA Biosciences, San Diego, CA, USA). Growth curves started to be automatically recorded in real-time on the xCELLigence System with a DP device. After cells proliferated until a cellular index (CI) over 1.0, usually after 24 h, the compounds studied were added, and growth curves were registered in real-time on a computer using RTCA 2.1.2. Software [65].

#### 3.4.9. Cytotoxicity Assay (MTS)

This study utilised an MTS-based colorimetric assay called CellTiter 96 Aqueous One Solution Cell Proliferation Assay (Promega, Madison, WI, USA) to measure cell viability. The assay was performed in triplicate in 96-well microtiter plates with flat bottom (Falcon). The method relies on the ability of metabolically active cells to reduce MTS, a yellow tetrazolium salt to the coloured formazan that is soluble in the culture medium. A total of 1 × 10^4^ cells/well were cultured in 100 μL for 24 h, culture supernatants were discarded, and then cells were treated for 24 and 48 h with increasing concentrations of drugs. After the end of the incubation time, 20 μL of reagent containing [3-(4,5-dimethylthiazol-2-yl)-5-(3-carboxymethoxyphenyl)-2-(4-sulfophenyl)-2H-tetrazolium, inner salt] (MTS) and PES (phenazine ethosulfate) was added in each well. Plates were incubated for 4 h at 37 °C, with mild agitation every 15 min. The reduction in the tetrazolium compound to formazan was spectrophotometrically measured at λ = 492 nm using a Dynex plate reader (DYNEX Technologies MRS, Chantilly, VA, USA). The percentage of viability compared to untreated cells (considered 100% viable) was calculated with the following formula:

Cell viability (%) = (absorbance of treated cells − absorbance of culture medium)/(absorbance of untreated cells − absorbance of culture medium) × 100.

The percentage of viability compared to untreated cells was calculated, and data were expressed as mean ± standard deviation (SD) of the experiments, obtained in triplicate (*n* = 3) [69].

#### 3.4.10. Apoptosis Assay Performed Using Flow Cytometry

The apoptosis assay was performed according to the manufacturer’s instructions using the Annexin V-FITC kit from BD Biosciences, San Jose, CA, USA. Cells treated and untreated with the experimental compound were resuspended in a cold binding buffer. The percentages of apoptotic cells were determined via double staining with Annexin V-FITC/PI. In each tube, 400 μL of Annexin V binding buffer was added, and the 5000 cells/sample were stored for 15 min in the dark at room temperature. The cells were analysed using a FACSCantoII flow-cytometer (Becton Dickinson-BD, Immunocytometry System, Mountain View, San Jose, CA, USA). The analysis was performed using DIVA 6.2 software to discriminate viable cells (FITC−PI−) from necrotic cells (FITC+PI+) and early apoptosis (FITC+PI−) from late apoptosis [66].

#### 3.4.11. Cell Cycle Analysis

In this experiment, 5 × 10^5^ cells that had been previously fixed were washed and resuspended in PBS. The CycleTEST PLUS DNA Reagent kit from BD Biosciences was used according to the manufacturer’s protocol to carry out the assay. The probes were kept in the dark and at 4 °C until data acquisition via flow cytometry (Becton Dickinson-BD, Immunocytometry System, Mountain View, San Jose, CA, USA) using a FACSCantoII flow-cytometer from BD. ModFIT v3.2 software was used to analyse the data and estimate the DNA index (DI) and progression through cell cycle phases [67].

#### 3.4.12. Statistical Analysis

All measurements were performed in triplicate. All data analyses were performed using GraphPad Prism 7 (GraphPad Software Inc., La Jolla, CA, USA). The differences between the treatment and control groups or between different treatments were statistically analysed using one-way analysis of variance ANOVA. Statistical significance was considered at *p* < 0.05.

## 4. Conclusions

Considering the world’s growing interest in natural health-promoting actives, the current study explored, for the first time, the combinatorial potential of *Sambucus nigra* and albumin-decorated–nanostructured lipid carriers as a new sustainable platform to support antitumour-targeted potential, particularly for the synergistic therapy of colon, ovarian, and/or breast cancer. The conventional NLC and hybrid albumin-NLC-*SambucusN* were built to host two lipophilic cores; one consisted of coconut butter, sage oil, and glycerol monostearate (NLC-I series), and the other consisted of methyl laurate associated with coconut butter and glycerol monostearate (NLC-II series). The average size for NLC-I-*SambucusN*-BSA was 140.2 ± 3.26 nm versus 143.9 ± 1.04 nm for NLC-II-*SambucusN*-BSA. A minimum polydispersity value of 0.120 ± 0.022 for NLC-I-*SambucusN-BSA* highlighted a relatively homogeneous and monodisperse distribution of nanocarriers. Coalescence and flocculation events were avoided by the creation of a surface layer with an electrokinetic zeta lesser than −50 mV. The capture of the *Sambucus nigra* extract was more efficient in NLC-I-*SambucusN* prepared with the lipid blend containing sage oil, i.e., an entrapment efficiency of 88.67 ± 4.75% *SambucusN* for NLC-I-*SambucusN* versus 74.49 V ± 6.70% for NLC-II-*SambucusN*. BSA-coated NLC-*SambucusN* resulted in a decrease in fluorescence due to changes in the environment of the albumin fluorophores, which occurred as a result of the creation of weak interactions between the functional groups of the phytochemicals extract and those of the albumin. 

The BSA-coated NLC-I/II-*SambucusN* showed free radical-scavenging activities when tested with an ABTS assay. The antioxidant activity of NLC-I/II-*SambucusN* was significantly enhanced using an albumin coating. The maximum annihilation of cationic radicals was 89.81 ± 4.84% and 86.01 ± 3.07% for NLC-I and II-*SambucusN* covered with a BSA biopolymer versus 74.47 ± 1.15% and 81.93 ± 2.70% determined for NLC-I and II-*SambucusN* without BSA. In addition, the IC_50_ value of 0.973 mg/mL revealed the superior antioxidant activity of NLC-II-*SambucusN*-BSA. According to the RTCA results, LoVo tumour colon cells were drastically affected by hybrid NLC; 100 µg/mL of NLC-I-*SambucusN*-BSA behaved similarly to the chemotherapeutic drug, cisplatin, with a cell viability of LoVo tumour cells of 21.81 ± 1.18%.

Albumin-decorated NLC-I and II-*SambucusN* ensured a certain intensity of the apoptosis process that caused the death of tumour cells in a high percentage, i.e., 50 μg/mL of NLC-II-*SambucusN*-BSA induced over 50% apoptosis in Lovo colon cells, 4 times higher compared to the untreated cells. A prolonged 48 h treatment with NLC-I-*SambucusN* appeared more efficient in the apoptosis of MCF-7 tumour breast cells, the apoptotic process being amplified by 5× compared to the control. The results obtained via flow cytometry show that the treatment of LoVo cells with NLC-II led to the blocking of the cell cycle in the G1 phase; this was accompanied by a significant decrease in the S phase. NLC-I and II-*SambucusN*-BSA were more efficient in the process of blocking the cell cycle in the G1 phase. Regarding the SKOV-3 ovarian cells, both lipid and hybrid protein–lipid nanocarriers acted in the sense of blocking the cell cycle in the G2 phase. The best efficiency in terms of total proliferation activity cessation and advanced induction of cell cycle arrest was determined for treatment with 50 µg/mL of NLC-I-*SambucusN* and NLC-II-*SambucusN*-BSA for 48 h.

The obtained result of the current study encourages the combinatorial potential of albumin-decorated–nanostructured lipid carriers loaded with *Sambucus nigra* as an effective platform that supports the destroying process of different tumour cells either via activating cell cycle-blocking mechanisms at the level of various restriction points that control the evolution of the cell cycle or by activating apoptotic pathways.

## Data Availability

The raw data supporting the conclusions of this article will be made available by the authors upon request.
